# Supplementary material for: The interactome of intact mitochondria by cross-linking mass spectrometry provides evidence for coexisting respiratory supercomplexes
Source: Mol Cell Proteomics. 2017 Dec 8;17(2):216–32. doi: 10.1074/mcp.RA117.000470 (PMC5795388; doi:10.1074/mcp.RA117.000470)
Supplement: Supplemental Data [file supp_RA117.000470_133922_0_supp_23978_fzffwf.zip › spectra_annotation/mito_DR_spectra_annotation/143-1-3-1-7-1.pdf]

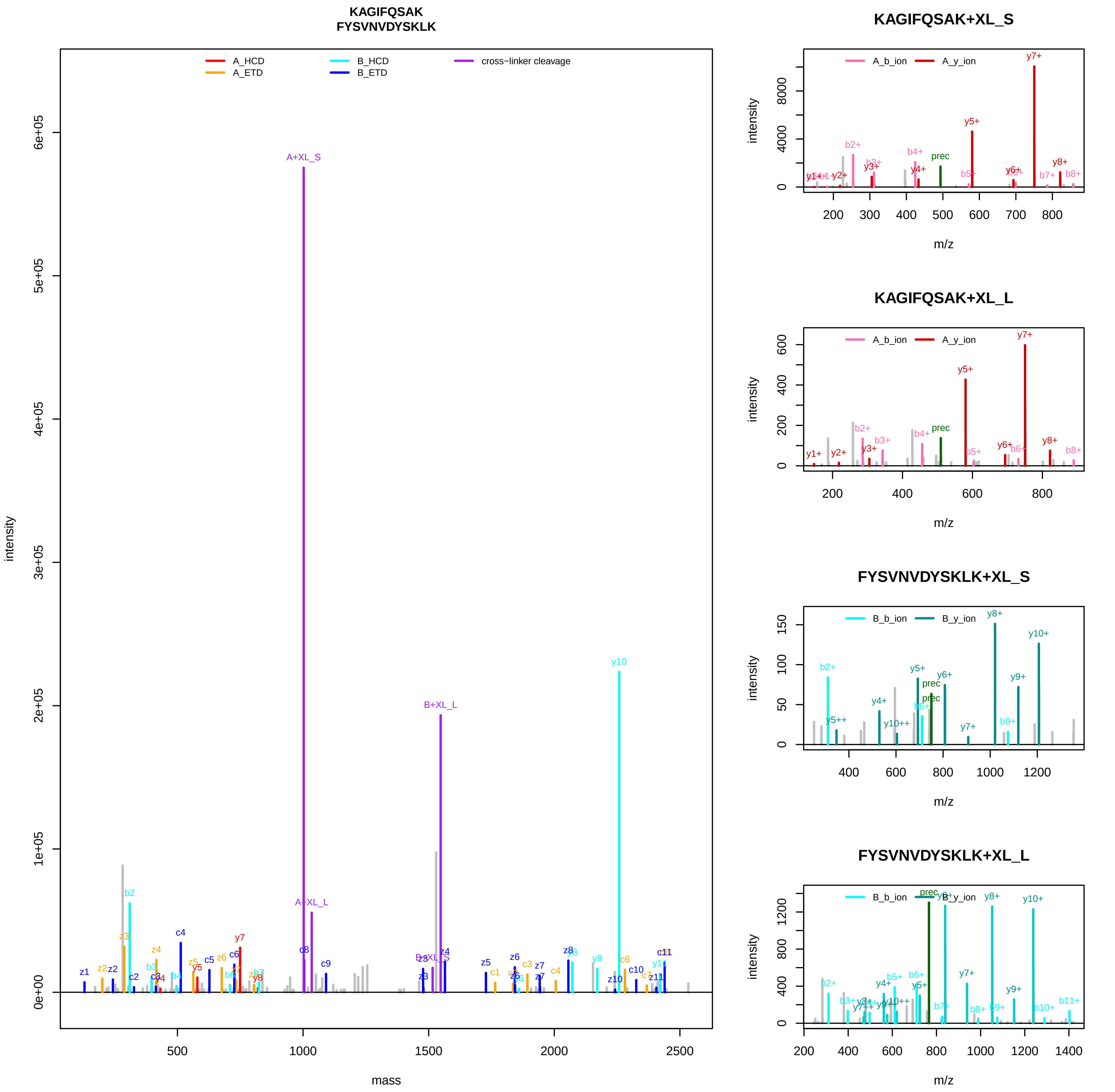
KAGIFQSAK
FYSVNVDYSKLK
A_HCD
A_ETD
B_HCD
B_ETD
cross−linker cleavage
intensity
mass
0e+00
1e+05
2e+05
3e+05
4e+05
5e+05
6e+05
500
1000
1500
2000
2500
A+XL_S
A+XL_L
B+XL_L
B+XL_S
y10
b2
z3
c4
y7
z4
c8
c9
z5
z6
c5
c6
c2
c3
z1
z2
y5
y8
z8
c10
z10
c11
z11
c7
c1
z7
b3
b4
b6
b7
y9
KAGIFQSAK+XL_S
A_b_ion
A_y_ion
intensity
m/z
0
4000
8000
200
300
400
500
600
700
800
y7+
y5+
b2+
b3+
b4+
prec
y3+
y4+
b5+
y6+
b6+
y8+
b7+
b8+
y1+
b1+
y2+
KAGIFQSAK+XL_L
A_b_ion
A_y_ion
intensity
m/z
0
200
400
600
200
400
600
800
y7+
y5+
b2+
b3+
b4+
prec
y1+
y2+
y3+
y6+
b5+
b6+
y8+
b8+
FYSVNVDYSKLK+XL_S
B_b_ion
B_y_ion
intensity
m/z
0
50
100
150
400
600
800
1000
1200
y8+
y10+
b2+
y5+
y6+
prec
y9+
y4+
b6+
y5++
y10++
y7+
b9+
FYSVNVDYSKLK+XL_L
B_b_ion
B_y_ion
intensity
m/z
0
400
800
1200
200
400
600
800
1000
1200
1400
prec
y6+
y8+
y10+
b2+
b3+
y4+
b5+
b6+
y5+
y7+
y9+
b7+
b8+
b9+
b10+
b11+
y10++
